# Remnant liver function is associated with long-term survival in patients with hepatocellular carcinoma undergoing hepatectomy

**DOI:** 10.1038/s41598-023-42929-x

**Published:** 2023-09-20

**Authors:** Atsushi Miki, Yasunaru Sakuma, Jun Watanabe, Kazuhiro Endo, Hideki Sasanuma, Takumi Teratani, Alan Kawarai Lefor, Atsushi Shimizu, Joji Kitayama, Yoshikazu Yasuda, Naohiro Sata

**Affiliations:** Division of Gastroenterological, General and Transplant Surgery, Department of Surgery, Jichi Medical University, 3311-1 Yakushiji, Shimotsuke, Tochigi 329-0498 Japan

**Keywords:** Outcomes research, Translational research, Risk factors

## Abstract

It is important to assess the prognosis and intervene before and after surgery in patients with hepatocellular carcinoma. This study aims to elucidate the association of outcomes and residual liver function after hepatectomy. A total of 176 patients who underwent the initial resection for hepatocellular carcinoma between January 2011 and March 2021 at Jichi Medical University were included. Hepatic clearance of the remnant liver was measured using 99mTc-galactosyl serum albumin scintigraphy. The log-rank test was used to analyze survival using the Kaplan–Meier method. Hazard ratios (HR) and 95% confidence intervals (CI) for overall survival were calculated using Cox’s proportional hazard model. In multivariate analysis, microvascular invasion, intraoperative blood loss, and hepatic clearance of the remnant liver were independently associated with overall survival. Hepatic clearance of the remnant liver was independently associated with recurrence free survival. This is the first report to show that lower residual liver function is associated with shorter survival in patients with hepatocellular carcinoma undergoing hepatectomy. Preoperative determination of remnant liver function may allow assessment of prognosis in patients planned to undergo resection of hepatocellular carcinoma. Preservation of liver functional reserve may be crucial for improved long-term outcomes after hepatectomy.

## Introduction

Hepatocellular carcinoma is the sixth most common cancer and the third leading cause of cancer-related deaths worldwide^1^. Patients with early stage hepatocellular carcinoma are ideal candidates for resection, in those patients with preserved liver function. Although improved diagnostic methods, surgical techniques, and perioperative management have led to better results^2^, the striking rate of recurrence after hepatectomy is still a barrier that diminishes the overall prognosis for these patients, with a cumulative recurrence rate of 50–60% at 3 years and 60–80% at 5 years^3^. As a result, there is an urgent need to determine how to predict prognosis and intervene as early as possible.

Pathologic factors associated with poor outcomes in patients with hepatocellular carcinoma have been studied, and some such as microvascular invasion, number of lesions and tumor size are considered to be associated with a poor prognosis. Biochemical factors, such as α-fetoprotein level, des-γ-carboxyprothrombin, indocyanine green retention 15 min (ICG-R15), and γ-glutamyl transpeptidase, have also been studied and used to predict tumor progression and prognosis^4^. The statistically derived albumin–bilirubin (ALBI) grade was proposed as a new assessment tool, and some studies of its usefulness have reported its ability to predict prognosis and assist in decision making regarding treatment choices for patients with hepatocellular carcinoma^5^.

In recent years, liver function evaluation methods using imaging data have been developed. 99mTc-galactosyl serum albumin (GSA) scintigraphy can assess liver function. GSA is a specifically recognized by asialoglycoprotein receptors on the surface of hepatocytes and is taken up by hepatocytes. Hepatic clearance estimated by GSA scintigraphy was reported to be a reliable index for accessing liver fibrosis^6^. In addition, residual liver function can be measured by GSA scintigraphy using hepatic clearance. We reported that hepatic clearance of the residual liver is associated with post-hepatectomy liver failure and postoperative complications^7^. These results suggested that the liver remnant is associated with short-term outcomes after hepatectomy in patients with hepatocellular carcinoma. However, little is known about the association of liver remnant function with long-term outcomes. The aim of this study is to elucidate the association of residual liver function with long-term outcome in patients with hepatocellular carcinoma undergoing hepatectomy.

## Results

### Patient characteristics

A total of 176 consecutive patients with hepatocellular carcinoma who underwent their initial resection were included with a median age of 70 years (range 24–87). Among the 176 patients, 95 (54%) had hepatitis C viral infections and 25 (14%) had hepatitis B viral infections. Most patients were Child–Pugh class A (174/177, 98%) and the remaining patients were class B (3/177, 2%). Pathologically, 50 (28%) patients had liver cirrhosis. With a median follow up of 47 months (range: 1–132), 87 patients (49%) developed recurrence of hepatocellular carcinoma and 51 patients (29%) died. The 5-year overall survival rate was 70.3% and 5-year recurrence free survival rate was 45.8%.

Platelet count, total bilirubin value, aspartate transferase value, γ-glutamyl transpeptidase value, ALBI score, number of intrahepatic tumors, undergoing perioperative blood transfusions, and intraoperative blood loss were significantly different between the group with an hepatic clearance in the remnant liver less than 205 ml/min and the group with more than 205 ml/min (Table 1).

**Table 1:** Patient characteristics according to hepatic clearance of the remnant liver.

Variables	Hepatic clearance < 205 ml/min (n = 71)	Hepatic clearance ≥ 205 ml/min (n = 105)	*p* value
Age (year) mean ± standard deviation	69.5 ± 8.7	68.3 ± 9.6	0.3865
Gender (female vs male)	19 vs 52	22 vs 83	0.3733
Platelet count (106/l) mean ± standard deviation	149	177	0.0074*
Total Bilirubin (mg/dl) mean ± standard deviation	0.92 ± 0.34	0.81 ± 0.38	0.0491*
AST (IU/l) mean ± standard deviation	55 ± 62	37 ± 20	0.0082*
ALT (IU/l) mean ± standard deviation	44 ± 34	38 ± 31	0.2187
γ-glutamyl transpeptidase (IU/l) mean ± standard deviation	100 ± 117	66 ± 62	0.0122*
AFP median (range) (ng/ml) mean ± standard deviation	4786 ± 27,547	3389 ± 15,167	0.6677
PIVKA II median (range) (mAU/ml) mean ± standard deviation	1440 ± 4250	1207 ± 3684	0.7062
ALBI score median mean ± standard deviation	− 2.54 ± 0.37	− 2.89 ± 0.31	0.0001*
Major surgery yes vs no	22 vs 49	8 vs 97	0.0001*
Tumor size median (mm) mean ± standard deviation	51.7 ± 33.6	45.6 ± 27.9	0.1964
Microvascular invasion yes vs no	30 vs 41	38 vs 67	0.4184
Liver fibrosis F1–3 vs F4	46 vs 25	81 vs 24	0.0744
Intrahepatic metastasis mean ± standard deviation	1.15 ± 0.47	1.26 ± 0.59	0.2224
Blood transfusion yes vs no	26 vs 45	17 vs 88	0.0024*
Intraoperative blood loss (ml) mean ± standard deviation	1181 ± 122	752 ± 886	0.0076*
Functional volume of the remnant liver (ml) mean ± standard deviation	838 ± 312	1162 ± 292	0.0001*
LHL15 median mean ± standard deviation	0.90 ± 0.04	0.093 ± 0.02	0.0001*

*AFP* alpha fetoprotein, *PIVKA II* protein induced by vitamin K absence, *ALBI* albumin bilirubin, *LHL15* liver to heart-plus-liver radioactivity at 15 min, *P < 0.05.

### Receiver operating characteristic curve analysis to determine the cut off value

**Figure 1:**
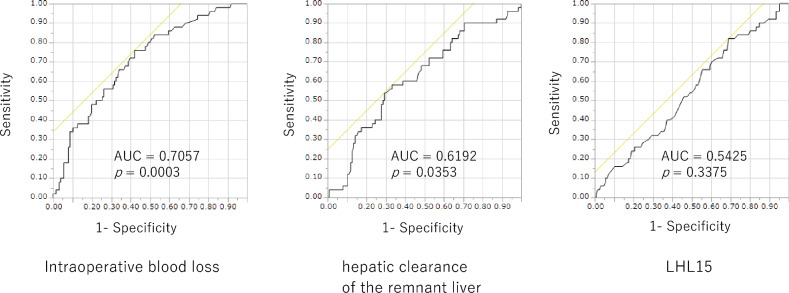
Receiver operating characteristics curve analysis. The cut-off values for intraoperative blood loss, hepatic clearance of the remnant liver, and liver to heart-plus-liver radioactivity at 15 min (LHL15) were 550 ml (AUC = 0.7057, *p* = 0.0003), 205 ml/min (AUC = 0.6192, *p* = 0.0353), and 0.92 (AUC = 0.5425, *p* = 0.3375). *AUC* area under the curve.

The cut-off values for intraoperative blood loss, hepatic clearance of the remnant liver, and liver to heart-plus-liver radioactivity at 15 min (LHL15) were 550 ml (AUC = 0.7057, *p* = 0.0003), 205 ml/min (AUC = 0.6192, *p* = 0.0353), and 0.92 (AUC = 0.5425, *p* = 0.3375) (Fig. 1).

### Factors associated with overall survival

In univariable analysis, γ-glutamyl transpeptidase (HR 1.74, *p* = 0.0495, 95% CI 1.001–3.073), alpha-fetoprotein (HR 1.81, *P* = 0.0352, 95% CI 1.042–3.169), tumor size (HR 1.91, *P* = 0.0212, 95% CI 1.102–3.351), stage (HR 2.22, *P* = 0.0074, 95% CI 1.229–4.152), microvascular invasion (HR 2.35, *P* = 0.0025, 95% CI 1.356–4.075), blood transfusion (HR 1.82, *P* = 0.0477, 95% CI 0.314–0.994), intraoperative blood loss (HR 2.26, *P* = 0.0083, 95% CI 1.234–4.134), and hepatic clearance of the remnant liver (HR 0.46, *P* = 0.0060, 95% CI 0.261–0.780) were significantly associated with overall survival. In multivariate analysis, intraoperative blood loss (HR 2.27, *P* = 0.0301, 95% CI 1.082–4.760), and hepatic clearance of the remnant liver (HR 0.52, *P* = 0.0381, 95% CI 0.279–0.965) were independent factors significantly associated with overall survival (Table 2).

**Table 2:** Analysis of factors associated with overall survival.

Variables	Univariate analysis			Multivariate analysis		
	HR	*p*	95% CI	HR	*p*	95% CI
Age (yr) > 70 years	1.25	0.4371	0.710–2.173			
Gender (male vs female)	1.10	0.7714	0.592–2.208			
Platelet count 106/l	1.11	0.8049	0.524–2.740			
Total Bilirubin > 1.5 mg/dl	1.07	0.9248	0.152–3.039			
AST > 37 IU/l	1.43	0.1971	0.828–2.476			
ALT > 44 IU/l	1.12	0.7079	0.631–2.072			
γ-glutamyl transpeptidase > 64 IU/L	1.74	0.0495*	1.001–3.073	1.12	0.7117	0.602–2.087
AFP > 10 IU/l	1.81	0.0352*	1.042–3.169	1.84	0.0626	0.969–3.561
PIVKA II > 40 IU/l	1.13	0.6739	0.646–2.014			
ALBI score > − 2.60	1.12	0.7032	0.620–1.959			
Major surgery yes vs no	2.26	0.0083*	1.234–4.142	1.43	0.3081	0.704–2.814
Tumor size > 5 cm	1.91	0.0212*	1.102–3.351	1.20	0.5623	0.643–2.264
Microvascular invasion yes vs no	2.35	0.0025*	1.356–4.075	1.88	0.0454*	1.013–3.510
Liver fibrosis F4 vs not	1.35	0.9125	0.574–1.960			
Intrahepatic metastasis	1.26	0.5450	0.403–1.751			
Blood transfusion yes vs no	1.82	0.0477*	0.314–0.994	1.01	0.9768	0.501–2.040
Intraoperative blood loss > 550 ml	2.26	0.0083*	1.234–4.134	2.28	0.0287*	1.095–4.856
Hepatic clearance of the remnant liver > 205 ml/min	0.46	0.0060*	0.261–0.780	0.52	0.0378*	0.277–0.964
LHL15 > 0.92	1.37	0.3132	0.750–2.687			

*HR* hazard ratio, *CI* confidence interval, *AFP* alpha fetoprotein, *PIVKA II* protein induced by vitamin K absence, *ALBI* albumin bilirubin, *LHL15* liver to heart-plus-liver radioactivity at 15 min, *P < 0.05.

### Factors associated with recurrence free survival

In univariable analysis, tumor size (HR 1.58, *P* = 0.0386, 95% CI 1.024–2.421), stage (HR 1.67, *P* = 0.0203, 95% CI 1.082–2.613), microvascular invasion (HR 1.63, *P* = 0.0299, 95% CI 1.050–2.504), liver fibrosis (HR 1.60, *P* = 0.0399, 95% CI 1.022–2.465), and hepatic clearance of the remnant liver (HR 0.59, *P* = 0.0141, 95% CI 0.383–0.897) were significantly associated with recurrence free survival. In multivariate analysis, hepatic clearance of the remnant liver (HR 0.63, *P* = 0.0398, 95% CI 0.411–0.979) was significantly associated with recurrence free survival (Table 3).

**Table 3:** Analysis of factors associated with recurrence free survival.

Variables	Univariate analysis			Multivariate analysis		
	HR	*p*	95% CI	HR	*p*	95% CI
Age (years) > 70 years	1.35	0.1693	0.878–2.072			
Gender (male vs female)	1.07	0.7919	0.665–1.789			
Platelet count 10^6^/l	0.74	0.3258	0.432–1.374			
Total Bilirubin > 1.5 mg/dl	1.62	0.2907	0.626–3.436			
AST > 37 IU/l	1.11	0.6212	0.725–1.697			
ALT > 44 IU/l	1.17	0.4991	0.539–1.332			
γ-Glutamyl transpeptidase > 64 IU/L	1.32	0.2096	0.851–2.036			
AFP > 10 IU/l	1.52	0.0530	0.995–2.334			
PIVKA II > 40 IU/l	1.14	0.5466	0.742–1.792			
ALBI score > − 2.60	1.53	0.0554	0.990–2.330			
Major surgery yes vs no	1.10	0.7494	0.617–1.957			
Tumor size > 5 cm	1.58	0.0386*	1.024–2.421	1.50	0.2493	0.751–3.014
Microvascular invasion yes vs no	1.63	0.0299*	1.050–2.504	1.43	0.2373	0.790–2.590
Liver fibrosis F4 vs not	1.60	0.0399*	1.022–2.465	1.50	0.0838	0.947–2.387
Intrahepatic metastasis	1.33	0.3243	0.448–1.346			
Blood transfusion yes vs no	1.57	0.0620	0.405–1.024			
Intraoperative blood loss > 550 ml	1.39	0.1326	0.904–2.147			
Hepatic clearance of the remnant liver > 205 ml/min	0.59	0.0141*	0.383–0.897	0.63	0.0398*	0.411–0.979
LHL15 > 0.92	0.74	0.1879	0.480–1.163			

*HR* hazard ratio, *CI* confidence interval, *AFP* alpha fetoprotein, *PIVKA II* protein induced by vitamin K absence, *ALBI* albumin bilirubin, *LHL15* liver to heart-plus-liver radioactivity at 15 min. *P < 0.05.

### Kaplan–Meier survival analysis based on hepatic clearance of the remnant liver, LHL15, and ALBI score

**Figure 2:**
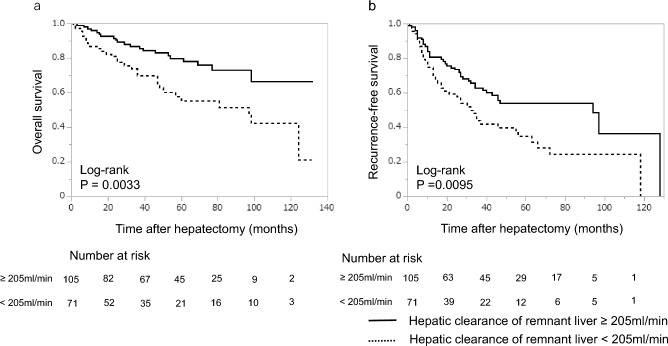
Kaplan–Meier curves for overall and recurrence-free survival associated with hepatic clearance of the remnant liver. (**a**) Overall survival comparing patients with hepatic clearance of the remnant liver < 205 ml/min and hepatic clearance of the remnant liver ≥ 205 ml/min. The overall survival in those with a hepatic clearance of the remnant liver ≥ 205 ml/min was significantly longer than that of those with clearance < 205 ml/min (*p* = 0.0033). (**b**) Recurrence free survival comparing patients with hepatic clearance of the remnant liver < 205 ml/min and hepatic clearance of the remnant liver ≥ 205 ml/min. The recurrence free survival of those with a hepatic clearance of the remnant liver ≥ 205 ml/min was significantly longer than that those with clearance < 205 ml/min (*p* = 0.0095).

The 5-year overall survival rate of patients with a hepatic clearance of the remnant liver less than 205 ml/min was 55.1% and those with a hepatic clearance of the remnant liver more than 205 ml/min was 79.9% (*P* = 0.0033) (Fig. 2a).

The 5-year recurrence free survival rate in patients with a hepatic clearance of the remnant liver less than 205 ml/min was 34.9% and those with a hepatic clearance of the remnant liver more than 205 ml/min was 53.9% (*P* = 0.0095) (Fig. 2b).

## Discussion

Many reports have demonstrated that residual liver function is associated with short-term outcomes, mainly post-hepatectomy liver failure, but no reports have discussed the impact of residual liver function on long-term outcomes. In the present study, independent prognostic factors significantly associated with overall survival were microvascular invasion, intraoperative blood loss and remnant liver function. The independent prognostic factor significantly associated with recurrence free survival was remnant liver function. The results of the present study show that measurement of remnant liver function may be useful to predict the overall and recurrence free survival in patients with hepatocellular carcinoma undergoing hepatectomy. Decreased intraoperative bleeding may result in improved survival. Owing to the high recurrence rate after hepatectomy, risk factors for tumor recurrence must be ascertained. That information could help be used to guide interventions earlier and facilitate better surveillance to reduce the rate of recurrence and improve the quality of care for patients with hepatocellular carcinoma.

There was not significant difference between insufficient hepatic clearance of remnant liver and both of tumor size (*p* = 0.3272) and microvascular invasion (*p* = 0.4177). Therefore advanced cancer situation may not be associated with insufficient hepatic clearance of the remnant liver. And insufficient hepatic clearance of the remnant liver was associated with tumor recurrence, therefore, insufficient hepatic clearance of the remnant liver itself may be driving prognostic risk. In the high clearance group, 32 patients (30%) underwent anatomical resection and in the low clearance group, 30 patients (42%) underwent anatomical resection (*p* = 0.1086). In our results, anatomical hepatic resections may not be associated with prolonged prognosis. Regardless of the surgical technique, the resulting remnant liver function may have an impact on prognosis.

Several methods of assessing residual liver function have been reported using computed tomography scan, magnetic resonance intensity, or GSA scintigraphy. Computed tomography scans clearly show the liver volume, but liver volume does not assess liver function. GSA scintigraphy and gadolinium ethoxybenzyldiethylenetriaminepentaacetic acid magnetic resonance intensity can quantitatively assess partial liver function and is useful for determining treatment intensity in patients with reduced hepatic reserve^8^. As a method to assess total liver function, the ICG test is the widely used throughout the world. ICG-R15 is reported to be an early indicator of hepatic dysfunction and has been used preoperatively to plan the extent of partial hepatectomy by predicting the risk of dysfunction after surgery. ICG-R15 together with γ-glutamyl transpeptidase was reported to be an independent preoperative factor associated with recurrence free survival^9^. However, because this method assumes the absence of interregional disparities of liver function, ICG-R15 cannot accurately predict residual liver function in the presence of interregional functional disparities due to impaired blood flow or biliary obstruction. Recently, the ALBI score has also been used as a prognostic factor. However, in the present study, the ALBI score is not associated with long-term outcomes in patients with hepatocellular carcinoma undergoing hepatectomy. In the present study, hepatic clearance of the remnant liver is associated with long-term outcomes. However, LHL15 expressing the whole liver function did not predict the long-term outcomes. Taken together, residual liver function may be more useful than total liver function in predicting long-term outcomes in patients with hepatocellular carcinoma undergoing hepatectomy.

A cut off value of < 205 ml/min clearance of the remnant liver was determined using the receiver operating characteristic curve method. The mean hepatic clearance of a normal liver is 400 ml/min ± 100 ml/min, so a clearance of 205 ml/min is approximately 2 standard deviations below the mean. This study shows that the prognosis of patients with a hepatic clearance of the remnant liver < 205 ml/min is poor. Further studies are needed to clarify the clinical significance of these values.

Massive hemorrhage remains a major complication of liver surgery. Increased blood loss during liver surgery has a significant impact on morbidity and mortality, and perioperative blood transfusions are associated with higher rates of postoperative complications and tumor recurrence^10,11^. Intraoperative blood loss was shown to result in immunosuppression by reducing natural killer cell activity and T-helper 1 lymphokines^12,13^. Many reports showed that blood transfusions negatively impact outcomes of hepatectomy. Devascularization techniques, hemostatic procedures, intraoperative ultrasound, and low central venous pressure inhalation have reduced intraoperative blood loss during hepatic resections^14–16^. Despite the availability of novel transection devices, vascular control is often required in complex hepatic procedures to avoid excessive bleeding^17^. Recently, laparoscopic hepatectomy has been introduced and has been shown to reduce blood loss, but increased long-term survival has not yet been reported^18,19^. The problem may not be due to the hemorrhage itself, but rather to a decrease in liver function due to liver damage caused by hemorrhage and ischemic-reperfusion injury, which may affect the liver function and prognosis. Therapeutic strategies tackling ischemic-reperfusion injury could not only improve post-surgical organ function, but also may reduce the risk of tumor recurrence^20^.

Microvascular invasion, reported in 15–57.1% surgical specimens of hepatocellular carcinoma^21^, is associated with aggressive biological features of hepatocellular carcinoma, which has been established as a risk factor for early recurrence and poor outcomes^22^. The exact biological mechanisms accounting for enhanced oncologic risk observed are not yet clear. Release of cancer cells by surgical manipulation with subsequent hematogenous spread could be one important means of metastasis formation^23^. To improve the prognosis of patients with hepatocellular carcinoma with microvascular invasion, anatomical subsegmentectomy or partial hepatectomy with a wide resection margin is recommended. In the present study, low hepatic clearance of the remnant liver was associated with shorter overall and recurrence free survival. Further study is needed to decide the optimal surgical margins.

Hepatocellular carcinoma patients with vascular invasion and cirrhosis have a high rate (78–83%) of developing recurrent disease within 6–35 months after resection^24^. In this study, the ratio that remnant liver clearance divided by total liver clearance was associated with micro vascular invasion (*p* = 0.0248) and tumor size (*p* = 0.0423). The ratio that remnant liver clearance divided by total liver clearance was associated with pathological findings and the remnant liver clearance. Therefore, the function of the remnant liver clearance may be associated with the recurrence of the primary tumor. The mechanism of the recurrence of the primary tumor is unclear. The possible explanation is that cirrhosis is associated with cytochrome p450 mediated drug clearance^25^. The down-regulation of the CYP2C19 gene were associated with the recurrence free survival in hepatocellular carcinoma^26^. CYP gene may be associated with the recurrence of the primary tumor. Further investigation is necessary to clarify the mechanism of the recurrence of primary tumor.

This study has several limitations. This is a retrospective study conducted at a single center with a relatively small sample size. If we use the value in clinical setting, we need another validation cohort. In this cohort, we do not have enough patient to validate. We need the further study to validate it in prospective multicenter trial. Potential confounding factors were assessed using descriptive statistics and univariable analysis and these factors were adjusted for whenever possible. Preoperative GSA scintigraphy was routinely performed to estimate total liver function. LHL15, remnant liver function, and laboratory data were objectively assessed in advance, which limited the risk of observation bias. The results presented should be prospectively validated in a larger population at multiple centers.

## Conclusion

To the best of our knowledge, this is the first report to show that low residual liver function is associated with shorter survival in patients with hepatocellular carcinoma undergoing hepatectomy. Preoperative measurement of remnant liver function may assist predicting prognosis in patients with hepatocellular carcinoma. Preservation of liver functional reserve may be crucial for improved long-term outcomes after hepatectomy in patient with hepatocellular carcinoma.

## Patients and methods

### Patients

A total of 176 patients who underwent the initial resection of hepatocellular carcinoma between January 2011 and March 2021 at Jichi Medical University were included in this study. There are no patients who died within 1 month after hepatectomy. All patients were regularly screened for recurrence using serum alpha-fetoprotein levels, protein induced by vitamin K absence II, and dynamic computed tomography (CT) scan every 3 months. The protocol for this study was approved by the Institutional Review Board and conforms to the provisions of the Declaration of Helsinki. All methods were carried out in accordance with relevant guidelines and regulations.

Blood samples obtained preoperatively were analyzed for routine liver tests. The cut-off values for intraoperative blood loss, hepatic clearance of the remnant liver, and liver to heart-plus-liver radioactivity at 15 min (LHL15) were determined by using a receiver operating characteristic curve analysis of overall survival (OS). The cut-off values for aspartate aminotransferase, alanine transaminase, γ-glutamyl transpeptidase, alpha-fetoprotein, and protein induced by vitamin K absence II were determined using hospital reference values. Recurrence was confirmed as local or distant by CT scan findings. For patients with recurrence, treatment was selected based on the performance status of the patient, liver function and extent of the recurrent tumors. OS was defined from the date of surgery to the date of death or last contact with the patient (censored). Recurrence-free survival was defined from the date of liver resection to the first recurrence at any site. Recurrence-free survival data were censored for patients who were alive without recurrence at the last follow-up date or who died without tumor recurrence. The tumor staging of patients with hepatocellular carcinoma followed the 8th edition of the American Joint Committee on Cancer staging system. The procedures for hepatectomy were categorized according to the Brisbane Nomenclature from the International Hepato-Pancreato-Biliary Association^27^. Briefly, the anatomic resection was defined as resection of the tumor together with the related portal vein branches and the corresponding hepatic territory. Major surgery was defined as hemihepatectomy and extended hemihepatectomy.

### Estimation of function of the remnant liver using GSA SPECT image

Patients underwent preoperative GSA scintigraphy as previously reported using a dual-head rotating gamma camera system and a dedicated data processing unit (Prism Axis, Picker Prism International, Cleveland, OH). A single bolus of 3 mg GSA (185 MBq; Nihon Medi-Phisics, Nishinomiya, Japan) was injected intravenously. After confirmation that the detector covered the area of the liver and heart, acquisition of planar images was begun with an acquisition time of 15 s each for 16 min immediately after injection. After acquisition of planar images, dynamic single photon emission tomography (SPECT) acquisition was started with an acquisition time of 20 s every 5 min. To generate a set of images equivalent to static SPECT images, projection data from dynamic SPECT were merged. Total liver function was calculated as the total liver GSA clearance, expressed in ml/min by the Patlak plot method. Regions of interest were generated for the entire liver on tomographic images using iso-count methods (25% cutoff of minimal count) to estimate the liver functional volume (ml). Hepatic clearance and functional volume of the remnant liver were estimated from fusion with CT scan images. Briefly, images from the CT scan were aligned with the image of the liver SPECT image with reference to the hepatic vein on every 3-mm liver cross-slice as a landmark on contrast-enhanced helical CT scan images. After the transection line had been set on the SPECT images based on the surgical procedure, the remnant liver with the resection line was determined manually. Remnant liver function was calculated from the proportional allocation of voxel count in static SPECT by the Patlak plot method and expressed by GSA clearance (ml/min). Regional functional liver volume (ml) was also calculated from the SPECT data by the outline extraction method.

### Statistical analysis

Continuous variables were presented as mean ± standard deviation and categorical variables are expressed as numbers. All categorical data were analyzed by Pearson’s chi-square test. Normally distributed values were analyzed by Student’s *t*-test. Non-normally distributed values were analyzed by the Mann–Whitney *U*-test. Overall survival curves were constructed using the Kaplan–Meier method. The log-rank test was used to analyze survival using the Kaplan–Meier method. Hazard ratios (HR) and 95% confidence intervals (CI) for OS were calculated using Cox’s proportional hazard model. All statistical analyses were performed using JMP version 16.0 (SAS Institute Inc., Cary, NC). The significance threshold was set at* p* < 0.05.

### Ethics approval statement

The study was reviewed and approved by the Institutional Review Board of Jichi Medical University, Approval No. A21-064.

### Patient consent statement

Written informed consent from any patient for data collection in a prospectively collected data base is available. However, the need for written informed consent for this study was waived by the Institutional Review Board of Jichi Medical University in view of the retrospective design of the study, based on national and local guidelines such as the fact that all clinical/laboratory measurements and procedures were part of routine care.

## Data Availability

The database contains highly confidential data which may provide insight in clinical and personnel information about patients and lead to their identification. Therefore, according to organizational restrictions and regulations these data cannot be made publicly available. However, the datasets used and/or analyzed during the current study are available from the corresponding author on reasonable request.
